# Species Origin of Genomic Factors in *Nicotiana nudicaulis* Watson Controlling Hybrid Lethality in Interspecific Hybrids between *N. nudicaulis* Watson and *N. tabacum* L

**DOI:** 10.1371/journal.pone.0097004

**Published:** 2014-05-07

**Authors:** Hongshuo Liu, Wataru Marubashi

**Affiliations:** Graduate School of Agriculture, Meiji University, Kanagawa, Japan; United States Department of Agriculture, United States of America

## Abstract

Hybrid lethality is expressed at 28°C in the cross *Nicotiana nudicaulis*×*N. tabacum*. The S subgenome of *N. tabacum* has been identified as controlling this hybrid lethality. To clarify the responsible genomic factor(s) of *N. nudicaulis*, we crossed *N. trigonophylla* (paternal progenitor of *N. nudicaulis*) with *N. tabacum*, because hybrids between *N. sylvestris* (maternal progenitor of *N. nudicaulis*) and *N. tabacum* are viable when grown in a greenhouse. In the cross *N. trigonophylla*×*N. tabacum*, approximately 50% of hybrids were vitrified, 20% were viable, and 20% were nonviable at 28°C. To reveal which subgenome of *N. tabacum* was responsible for these phenotypes, we crossed *N. trigonophylla* with two progenitors of *N. tabacum*, *N. sylvestris* (SS) and *N. tomentosiformis* (TT). In the cross *N. sylvestris*×*N. trigonophylla*, we confirmed that over half of hybrids of *N. sylvestris*×*N. trigonophylla* were vitrified, and none of the hybrids of *N. trigonophylla*×*N. tomentosiformis* were. The results imply that the S subgenome, encoding a gene or genes inducing hybrid lethality in the cross between *N. nudicaulis* and *N. tabacum*, has one or more genomic factors that induce vitrification. Furthermore, in vitrified hybrids of *N. trigonophylla*×*N. tabacum* and *N. sylvestris*×*N. trigonophylla*, we found that nuclear fragmentation, which progresses during expression of hybrid lethality, was accompanied by vitrification. This observation suggests that vitrification has a relationship to hybrid lethality. Based on these results, we speculate that when *N. nudicaulis* was formed approximately 5 million years ago, several causative genomic factors determining phenotypes of hybrid seedlings were inherited from *N. trigonophylla*. Subsequently, genome downsizing and various recombination-based processes took place. Some of the causative genomic factors were lost and some became genomic factor(s) controlling hybrid lethality in extant *N. nudicaulis*.

## Introduction

Hybrid lethality is a postzygotic barrier of reproductive isolation, which can occur in intergeneric, interspecific, and intraspecific crosses and causes death of some hybrids in *Nicotiana* species, *Arabidopsis thaliana*, tomato (*Solanum pimpinellifolium*×*S. lycopersicum*) and lettuce (*Lactuca saligna*×*L. sativa*) [1]–[5]. Bomblies and Weigel [6] concluded that hybrid lethality is a barrier to gene flow in *Arabidopsis thaliana* and tomato (*Solanum pimpinellifolium*×*S. lycopersicum*) and acts as an autoimmune-like response. Hybrid lethality is a mechanism playing a crucial role in the evolution of plants and is an important obstacle in plant breeding programs. In *Nicotiana*, hybrid lethality shows five specific phenotypes depending on the cross combination [7], [8].

The genus *Nicotiana* (Solanaceae) includes 76 species that are classified into 12 sections distributed mainly in the Americas and one section found outside the Americas; this section is *Suaveolentes*, which is restricted to Australia, islands of the South Pacific, and Africa [9]. The section *Suaveolentes* includes 26 species, 20 of which produce nonviable hybrids in crosses with well-studied cultivated tobacco lines belonging to section *Genuinae*, namely *Nicotiana tabacum* (2n = 48, SSTT), which originated by interspecific hybridization of *N. sylvestris* (2n = 24, SS) with *N. tomentosiformis* (2n = 24, TT) followed by chromosome doubling [10]–[17].

In crosses between 11 of the 20 *Suaveolentes* species and *N. tabacum*, the S subgenome of *N. tabacum* is involved in hybrid lethality at 28°C [18]–[23]. Furthermore, causative gene(s) of hybrid lethality in *N. tabacum* are encoded on the Q chromosome belonging to the S subgenome [24]–[28]. Based on these results, Tezuka [2] speculated that many species of section *Suaveolentes* share the same gene(s) triggering hybrid lethality by interaction with gene(s) on the Q chromosome. Recently, the first linkage map for *N. tabacum* was constructed by Bindler et al. [29] with SSR markers. Tezuka et al. [30] used this map to determine that the gene(s) responsible for hybrid lethality map to the region between SSR markers PT30342 and PT30365 of linkage group 11, corresponding to the Q chromosome.

Although investigations into the causative gene(s) in *N. tabacum* for hybrid lethality have been extensive, investigations on genomic factor(s) in wild species of section *Suaveolentes* have not. Although a single dominant gene, designated *HYBRID LETHALITY A1* (*HLA1*), controls hybrid lethality in *N. debneyi* (a wild species of section *Suaveolentes*) by interaction with gene(s) on the Q chromosome of *N. tabacum*, the gene location is unknown [31].

Section *Repandae* exists in North America and consists of four allopolyploid species: *N. nudicaulis*, *N. repanda*, *N. stocktonii* and *N. nesophila*. Section *Repandae* is considered to have originated approximately 5 million years ago from hybridization between ancestors of extant species of *Nicotiana* section *Sylvestres* (*N. sylvestris*) and *Trigonophyllae* (*N. obtusifolia*, formerly known as *N. trigonophylla*), the maternal and paternal progenitors, respectively, and their evolutionary relationships are well known [17], [32], [33]. *N. nudicaulis* is sister to, and morphologically distinct from, the three remaining species, diverging from them 2–3 million years ago, whereas *N. stocktonii* and *N. nesophila* diverged from *N. repanda* one million years ago; section *Repandae* is genetically distant from *N. tabacum*, even though they share the same maternal progenitor, *N. sylvestris* [34], [35].

In a previous study [36], hybrid lethality was observed in crosses of *N. repanda*×*N. tabacum*. Furthermore, Kobori and Marubashi [37] concluded that the T subgenome of *N. tabacum* encodes the causative gene(s) for hybrid lethality in the cross *N. repanda*×*N. tabacum*. On the other hand, the S subgenome of *N. tabacum* is related to hybrid lethality at 28°C in hybrids between *N. nudicaulis* and *N. tabacum* [38], [39]. In contrast to research on the subgenome controlling hybrid lethality in *N. tabacum*, no studies of the subgenome of wild species involved in hybrid lethality have been reported to our knowledge. In previous studies [40], [41], *N. sylvestris* (maternal progenitor of *N. nudicaulis*) was crossed with *N. tabacum*, and viable hybrids were produced. Here, we added the cross between *N. trigonophylla* (paternal progenitor of *N. nudicaulis*) and *N. tabacum* to infer the likely origins of genomic factor(s) in this wild species.

Apoptosis is a distinct type of cell death, and it is characterized in animals by condensation of chromatin, fragmentation of nuclei, cytoplasmic reduction, and fragmentation of DNA [42]–[44]. In hybrid seedlings of *N. nudicaulis*×*N. tabacum* expressing hybrid lethality, features of apoptosis such as chromatin condensation, nuclear fragmentation, and fragmentation of DNA were evident [38].

In the present study, we examined several phenotypes of hybrid seedlings in the cross *N. trigonophylla*×*N. tabacum*, and half of the hybrid seedlings showed vitrification. Vitrification frequently occurs and is a serious problem in plant micropropagation. Vitrified plants have translucent stems, brittle, water-soaked, elongated and curled leaves [45]. This report is the first to confirm that vitrified hybrid seedlings appear in interspecific crosses and that nuclear fragmentation is typically associated with vitrification. Because nuclear fragmentation also progresses during expression of hybrid lethality in the cross between *N. nudicaulis* and *N. tabacum* [38], we concluded that there is a relationship between vitrification and hybrid lethality. Furthermore, we found that the causative gene(s) in *N. tabacum* of vitrification are encoded on the S subgenome involved in hybrid lethality. These results also suggest that vitrification has a relationship to hybrid lethality. On the other hand, when *N. sylvestris* was crossed with *N. tabacum*, hybrid lethality was not observed [41], [42]. Based on these results, we inferred that the genomic factor(s) in *N. nudicaulis* leading to hybrid lethality in the cross between *N. nudicaulis* and *N. tabacum* is likely derived from *N. trigonophylla*.

## Materials and Methods

### Plant Materials

The seeds of *N. trigonophylla* Dunal (2n = 24, TrTr), *N. tabacum* (2*n* = 48, SSTT) ‘Red Russian,’ *N. sylvestris* Speg. & Comes (2n = 24, SS) and *N. tomentosiformis* Goodsp. (2n = 24, TT) were used in these experiments and were supplied by Japan Tobacco Inc. (Oyama, Japan). Plants were grown and pollinated in a greenhouse of the School of Agriculture, Meiji University.

### Ovule Culture

Because in conventional crossing between *N. trigonophylla* and *N. tabacum*, it is difficult to obtain hybrid seedlings, ovule culture was carried out 5 days after pollination and seedlings obtained as described by Chung et al. [46]. Flowers of *N. trigonophylla* used as the maternal parent were emasculated before flowering and pollinated with fresh *N. tabacum* pollen. Five days after pollination, flowers of *N. trigonophylla* were collected and the sepals, petals, and styles were removed. The ovaries were surface-sterilized with 70% ethanol for 30 s with 5% sodium hypochlorite for 10 min, then rinsed three times with sterilized water. The ovary walls were peeled to expose the placenta with intact ovules. All of the ovules were excised and cultured on Petri dishes containing 8 ml 1/2x MS medium [47] supplemented with 8% sucrose and 0.25% Gelrite, pH 5.8, at 28°C under continuous illumination (30 µmol photons s^−1^ m^−2^).

### Test Tube Pollination and Ovule Culture

Because no fertile seeds were obtained *in situ* from the cross *N. trigonophylla*×*N. sylvestris* or *N. sylvestris*×*N. trigonophylla*, and ovaries of *N. trigonophylla* are too small for test tube pollination and ovule culture, we carried out test tube pollination and ovule culture in the cross *N. sylvestris*×*N. trigonophylla* as described [48], and hybrid seedlings were obtained.

### Cultivation of Hybrid Seedlings

Immediately after germination, all hybrid seedlings of *N. trigonophylla*×*N. tabacum* or *N. sylvestris*×*N. trigonophylla* were separately transferred onto the surface of 1/2x MS medium with 1% sucrose at 28°C under continuous illumination (123 µmol s^−1 ^m^−2^ or 147 µmol s^−1 ^m^−2^). Some hybrid seedlings were transferred to flat-bottomed test tubes (25 mm diameter, 100 mm length) that contained 10 ml 1/2x MS medium supplemented with 1% sucrose and 0.25% Gelrite, pH 5.8, 20 days after germination (DAG).

### Interspecific Crosses of *N. trigonophylla*×*N. tomentosiformis*

Conventional crossing was carried out as follows: flowers of *N. trigonophylla* used as the maternal parent were emasculated before flowering and then pollinated with fresh *N. tomentosiformis* pollen. F1 seeds (*N. trigonophylla*×*N. tomentosiformis*) were soaked in 0.05% gibberellic acid (GA_3_) solution for 30 min, sterilized with 5% sodium hypochlorite for 15 min and then rinsed three times with sterilized water. Sterilized F1 seeds were sown on Petri dishes containing 8 ml 1/2x MS medium [47] supplemented with 1% sucrose and 0.25% Gelrite, pH 5.8; the plates were maintained for seed germination at 28°C under continuous illumination (30 µmol s^−1 ^m^−2^). Hybrid seedlings cultured at 28°C were all viable. These seedlings were potted at 20 DAG and cultivated in a greenhouse under natural lighting conditions.

### Random Amplified Polymorphic DNA (RAPD) Analysis

Total DNA was extracted from the leaves of the hybrid seedlings using cetyltrimethylammonium bromide [49]. RAPD analysis was carried out as described [39] using 20 random 10-mer oligonucleotide primers (Kit OPE, OPF; Operon Technologies, Inc., Alameda, CA, USA). PCR amplification was performed using an Applied Biosystems 2720 Thermal Cycler (Applied Biosystems, Foster City, CA, USA) programmed for 3 min at 94°C for initial denaturation, followed by 60 cycles of 30 s at 94°C, 1 min at 28°C for seedlings of *N. trigonophylla*×*N. tomentosiformis*, 32°C for seedlings of *N. trigonophylla*×*N. tomentosiformis*, or 25°C for seedlings of *N. sylvestris*×*N. trigonophylla*, then 2 min at 72°C, and a final extension of 5 min at 72°C. PCR products were separated by electrophoresis on 1% agarose gels in TAE buffer and stained with ethidium bromide to visualize DNA bands.

### Chromosome Analysis

Root tips of viable seedlings were pretreated with distilled water for 24 h at 4°C and with 2 mM 8-hydroxyquinoline for 4 h at 4°C, then fixed in a 3∶1 mixture of ethanol and acetic acid overnight to determine chromosome numbers. The root tips were hydrolyzed in 1 N HCl for 9 min at 60°C, stained with Schiff’s reagent and squashed in 45% acetic acid. The number of chromosomes in at least five root tip cells for each plant was counted under a BX51 light microscope (Olympus, Tokyo, Japan), and photographed using a DP70 automatic photomicrography system (Olympus).

### Flow Cytometry

For cytometric analysis, nuclei were isolated from 100 mg leaves (except for midrib) of *N. trigonophylla*×*N. tabacum* or *N. sylvestris*×*N. trigonophylla* hybrid seedlings at 28°C; the leaves were chopped off and macerated in ice-cold buffer [50]. Four plants were analyzed for each type of sample. The macerated tissue was filtered through a 25 µm nylon mesh. Nuclei were collected from the filtrate by centrifugation (5 min at 3000 rpm and 4°C) and suspended in ice-cold buffer supplemented with 5 µl/ml DAPI for 1 min at 4°C. The DNA content of the isolated nuclei was analyzed by flow cytometry on a Cell Lab Quanta SC system (Beckman Coulter Inc., La Brea, CA, USA). Between 16,000 and 22,000 nuclei were counted.

## Results

### Types of Abnormal Hybrid Seedlings of *N. trigonophylla*×*N. tabacum* Observed

When the cross *N. trigonophylla*×*N. tabacum* was carried out via conventional cross-pollination, no hybrid seedlings were obtained. Ovule culture was needed, as described previously [46]. Therefore, flowers of *N. trigonophylla* were pollinated with fresh *N. tabacum* pollen. From 31 flowers, 8284 ovules were obtained 5 days after pollination and 92 began to germinate 1 month after pollination. To confirm the expression of hybrid lethality, all of these seedlings were left at 28°C (Table 1).

**Table 1:** Interspecific hybridization of *N. trigonophylla*×*N. tabacum*.

				No. of hybrids				
Cross combination	No. of flowerspollinated	No. of ovulescultured	No. of hybridsobtained	Vitrified	Nonviable	Viable	Tumorous	Malformed
*N. trigonophylla*×*N. tabacum*	31	8284	92	43	24	21	2	2

**Figure 1:**
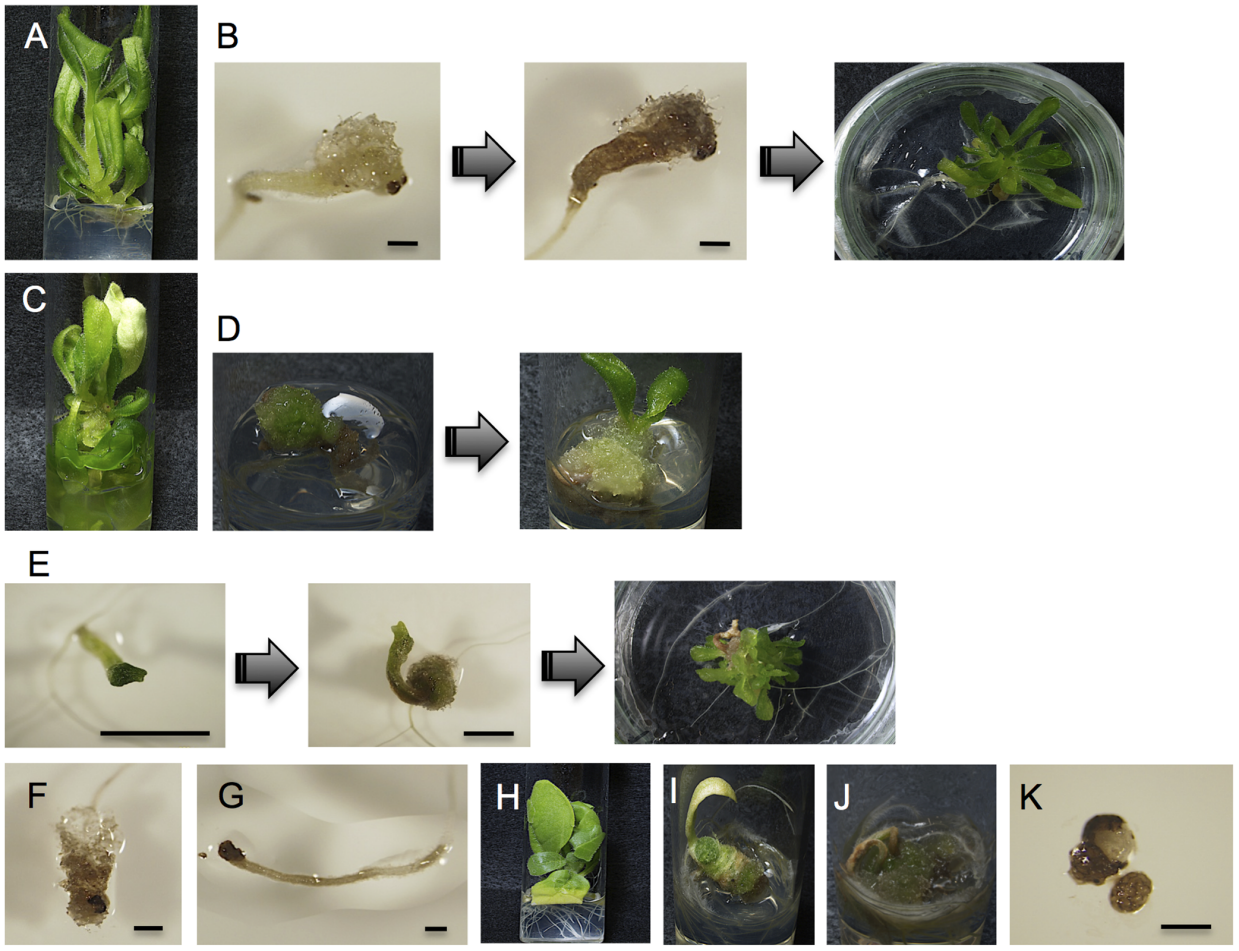
Several types of abnormal hybrid plants from *N. trigonophylla*×*N. tabacum* cultured at 28°C. (A) A vitrified hybrid plant. (B) A vitrified hybrid plant that changed from a browning tumorous plant. (C) A vitrified hybrid plant coexisting with tumors. (D) Vitrification that occurred on a tumorous hybrid plant. (E) A vitrified hybrid plant that changed from a tumorous plant, which had developed at the crown of a malformed plant. (F) A tumorous hybrid plant that died 10 DAG. (G) A malformed hybrid plant that died 14 DAG. (H) A viable hybrid plant coexisting with tumors. (I) A tumorous hybrid plant that developed at the crown of a yellowing plant. (J) A tumorous hybrid plant that developed at the crown of a browning vitrified plant. (K) A malformed hybrid plant. Scale bars are 1 mm.

Of the seedlings obtained, 43 were vitrified, 24 were nonviable, 21 were viable, 2 were tumorous hybrids, and 2 showed malformation (Figure 1).

Of the vitrified 43 hybrid seedlings, 25 hybrid plants were vitrified after germination (Figure 1A). Eight hybrid plants were tumorous after germination, and became vitrified after browning (Figure 1B). Seven vitrified hybrid plants coexisted with tumors after germination (Figure 1C). Two hybrid plants were tumorous after germination, and then became vitrified (Figure 1D). One hybrid plant was malformed after germination. It developed a tumor at the crown, and then became vitrified (Figure 1E).

Of the 24 nonviable hybrid seedlings, 20 were tumorous after germination and died 15 DAG (Figure 1F). The others were malformed after germination, and died 7 DAG (Figure 1G). On the other hand, of the 21 viable hybrid seedlings, six coexisted with tumors after germination (Figure 1H).

Of the two tumorous hybrid seedlings, one had a tumor that developed at the crown of the hybrid seedling, which was yellowing (Figure 1I). The other tumor developed at the crown of the seedling, which was browning and vitrified (Figure 1J).

**Figure 2:**
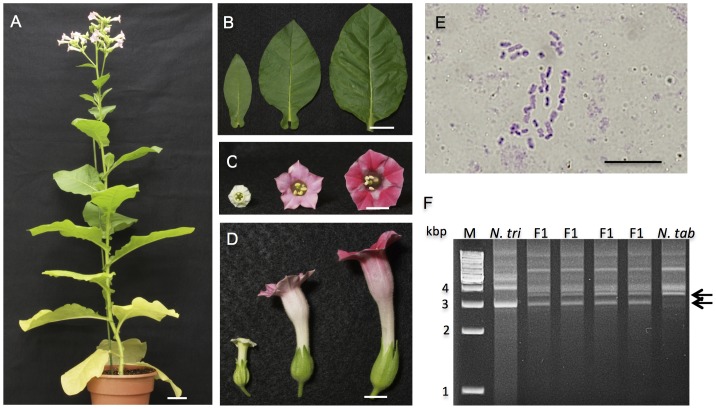
A hybrid of *N. trigonophylla*×*N. tabacum*. (A) Shape of a hybrid plant that grew to maturity and flowered. (B) Leaves of *N. trigonophylla*, a hybrid and *N. tabacum* (left to right). (C and D) Flowers of *N. trigonophylla*, a hybrid and *N. tabacum* (left to right). (E) Image of root tip cell of a hybrid plant, showing the number of chromosomes. (F) Confirmation of hybrid formation by RAPD analysis using primer OPE-14. The bands characteristic of both parents are indicated by arrows. M, size marker OneSTEP Ladder 1kb (1–10 kbp). Scale bars are 10 cm (A), 3 cm (B), 1 cm (C and D) and 100 µm (E).

To determine whether the viable and vitrified seedlings were true hybrids, we carried out morphological observation, RAPD analysis and chromosomal analyses on the viable seedlings, and flow cytometry on the vitrified seedlings. Four randomly selected seedlings were cultivated in a greenhouse for 60 DAG. All of the selected seedlings grew to maturity and came into flower (Figure 2A). The mature hybrid plants displayed uniform morphological characteristics, with leaf and flower shapes that were intermediate in appearance between those of the parents (Figure 2B-D). Chromosomal analyses of the hybrid plants revealed that each possessed 36 chromosomes, which is the sum of the number of haploid chromosomes of the parents (Figure 2E).

RAPD analysis using the primer OPE-14 was carried out on four viable seedlings. In these plants, several bands characteristic of both parents coexisted (Figure 2F, arrows); these patterns indicated that they are true hybrids. Similar results, shown in Figure 2F, were also obtained using several other random primers. All of the viable hybrid seedlings from the cross *N. trigonophylla*×*N. tabacum* were male sterile.

**Figure 3:**
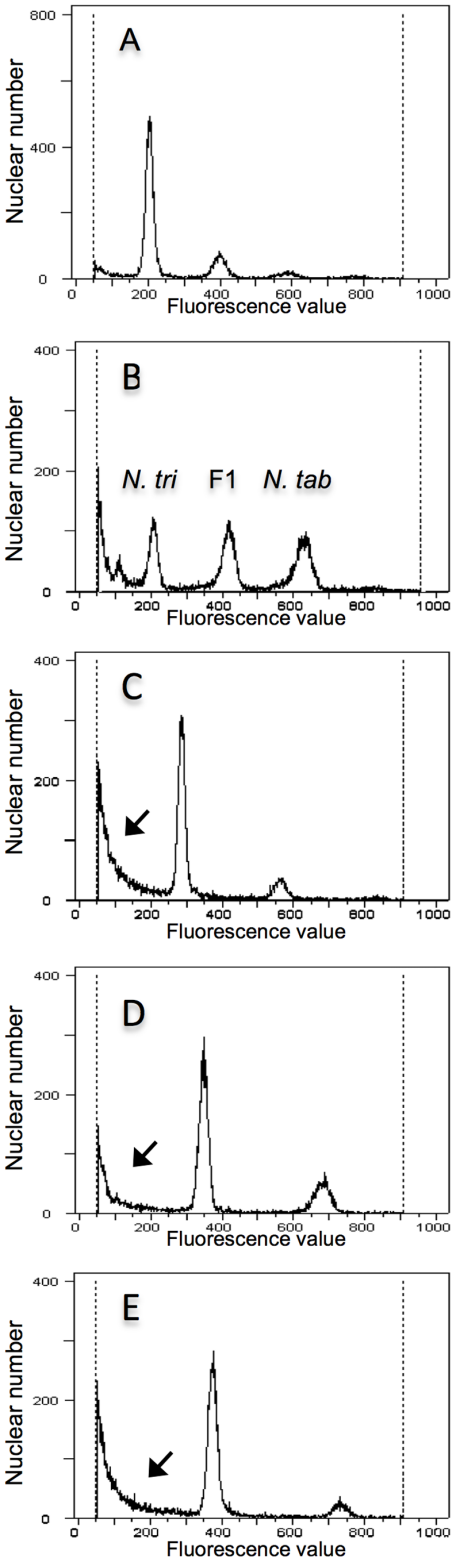
Histograms indicating hybridity for hybrid seedlings of *N. trigonophylla*×*N. tabacum*. The DNA content of 16,000–22,000 nuclei was determined by flow cytometry. (A) A sample of leaf cells from *N. trigonophylla* as the internal standard. The two peaks are characteristic of G1 nuclei and G2/M nuclei. (B) The G1 peak of a viable hybrid plant was intermediate between the two parents. The abbreviations *N. tri* and *N. tab* are for *N. trigonophylla* and *N. tabacum,* respectively. (C, D and E) The G1 peaks of vitrified hybrid seedlings were also intermediate between parents. However, the DAPI fluorescence values of these G1 peaks were closer to that of *N. trigonophylla* and the fluorescence values of G1 peaks differed among vitrified hybrid plants. Furthermore, subG1 peaks (black arrows), indicating nuclear fragmentation, with lower fluorescence values than that of G1 peaks appeared.

Flow cytometric analysis showed that the vitrified plants were true hybrids (Figure 3). The DAPI fluorescence values of the peaks corresponding to the G1 phase of the cell cycle differed, with values increasing in the order *N. trigonophylla*, viable hybrids and *N. tabacum*, using the G1 peak of *N. trigonophylla* as the internal standard in this experiment. The G1 peak of a viable hybrid was intermediate between the values of each parent (Figure 3B). The G1 peak values of vitrified seedlings were also intermediate between that of the parents. However, fluorescence values of these G1 peaks were closer to that of *N. trigonophylla* and were different from that of viable hybrid seedlings. Furthermore, subG1 peaks, with lower fluorescence values than that of G1 peaks, indicating nuclear fragmentation, appeared and the values for G1 peaks differed among vitrified hybrid plants (Figure 3C-E, black arrows).

### Types of Abnormal Hybrid Seedlings of *N. sylvestris*×*N. trigonophylla* Observed

Reciprocal crosses between *N. trigonophylla* and *N. sylvestris* were carried out using conventional cross-pollination, but no hybrid seedlings were obtained (Table 2). Therefore, test tube pollination and ovule culture were performed to overcome the crossing barrier.

**Table 2:** Interspecific hybridization between *N. trigonophylla* and *N. sylvestris*.

				No. of hybrids		
Cross combination	No. of flowers/placentas pollinated	No. of ovulescultured	No. of hybridsobtained	Vitrified	Viable	Nonviable
*N. trigonophylla*×*N. sylvestris*	10	–	–	–	–	–
*N. sylvestris*×*N. trigonophylla*	129	634	147	89	57	1

**Figure 4:**
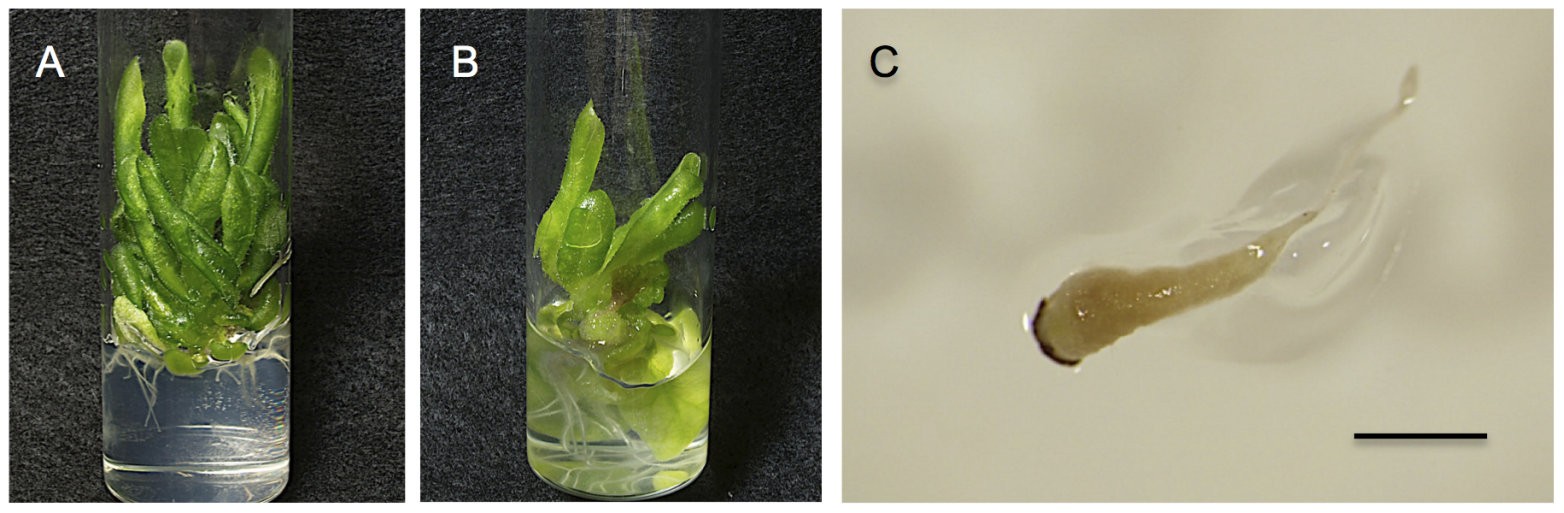
Several types of abnormal hybrid plants from *N. sylvestris*×*N. trigonophylla* cultured at 28°C. (A) A vitrified hybrid plant. (B) A vitrified hybrid plant coexisting with tumors. (C) A malformed hybrid plant that died 3 weeks after germination. Scale bar is 1 mm.

Pollination of 129 placentas of *N. sylvestris* in vitro resulted in 634 enlarged ovules that were cultured at 28°C, and 147 hybrid seedlings germinated from these ovules. Vitrified, viable and nonviable hybrid seedlings were observed; there were 89 vitrified seedlings, 57 viable seedlings and one nonviable seedling (Table 2). Of the 89 vitrified seedlings, 32 coexisted with tumors (Figure 4A and B). The growth of one nonviable hybrid seedling stopped at an early stage, and it died 60 DAG (Figure 4C).

**Figure 5:**
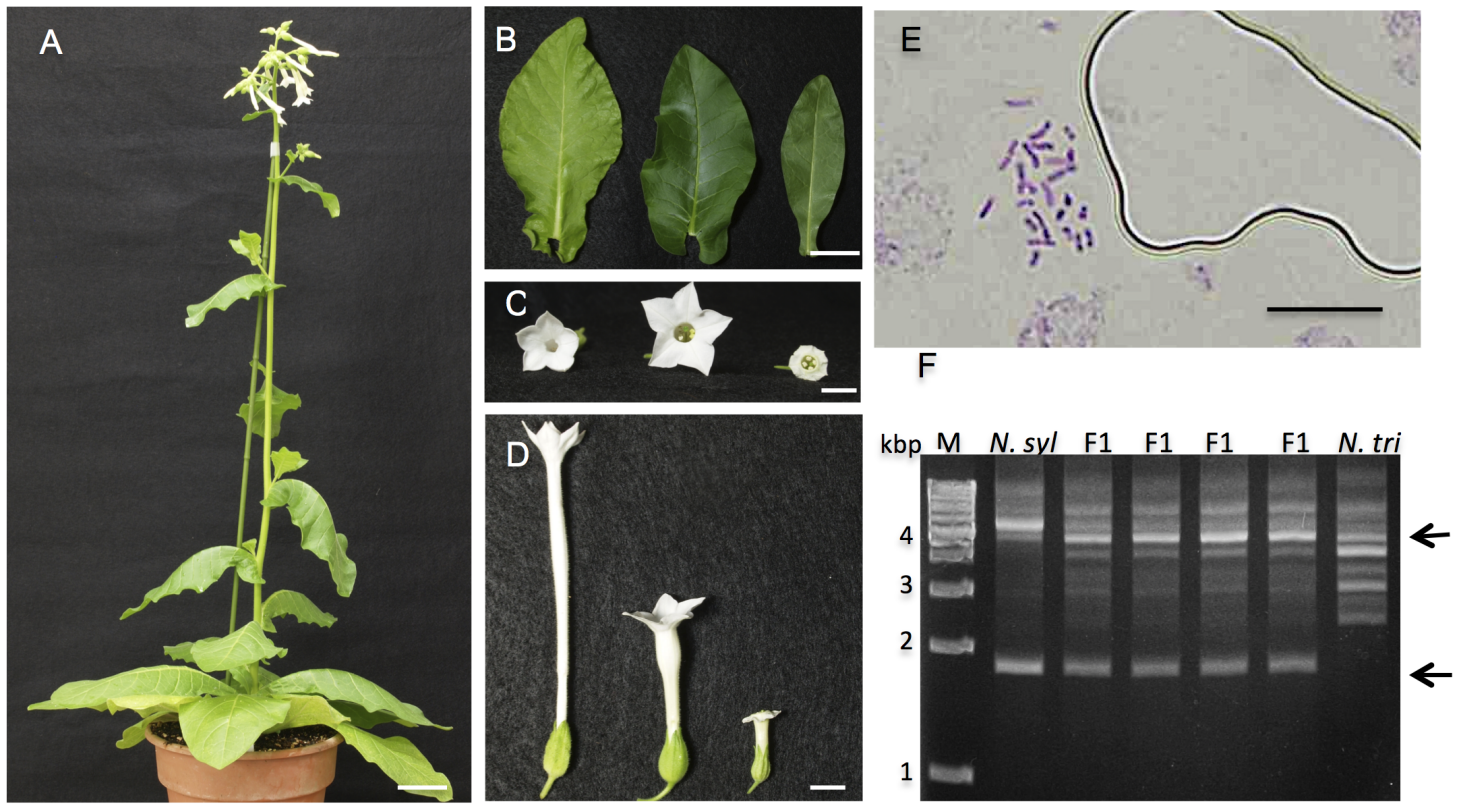
A hybrid of *N. sylvestris*×*N. trigonophylla*. (A) Shape of a hybrid plant that grew to maturity and flowered. (B) Leaves of *N. trigonophylla*, a hybrid and *N. tabacum* (left to right). (C and D) Flowers of *N. trigonophylla*, a hybrid and *N. tabacum* (left to right). (E) Image of root tip cell of a hybrid plant, showing the number of chromosomes. (F) Confirmation of hybrid formation by RAPD analysis using primer OPF-03. The bands characteristic of both parents are indicated by arrows. M, size marker OneSTEP Ladder 1kb (1–10 kbp). Scale bars are 10 cm (A), 3 cm (B), 1 cm (C and D) and 100 µm (E).

To confirm that these seedlings were true hybrids, morphological observation, RAPD analysis and chromosome analysis were carried out for the four viable seedlings, and flow cytometry was carried out for the vitrified seedlings. All of the viable hybrid seedlings grew to maturity and flowered (Figure 5A). The leaf shape, flower shape and flower color of the viable hybrid seedlings were intermediate in appearance between that of the parents (Figure 5B-D). Four hybrid plants, selected randomly, had 24 chromosomes (Figure 5E), which is the sum of the number of haploid chromosomes of their parents, *N. sylvestris* (2n = 24) and *N. trigonophylla* (2n = 24). These hybrid plants were male sterile. *N. sylvestris*, *N. trigonophylla*, and hybrid plants were also analyzed using RAPD. Hybrid plants had several bands in common between the RAPD patterns of parents using several primers (data not shown). RAPD patterns obtained with the primer OPF-03 are shown in Figure 5F as an example.

**Figure 6:**
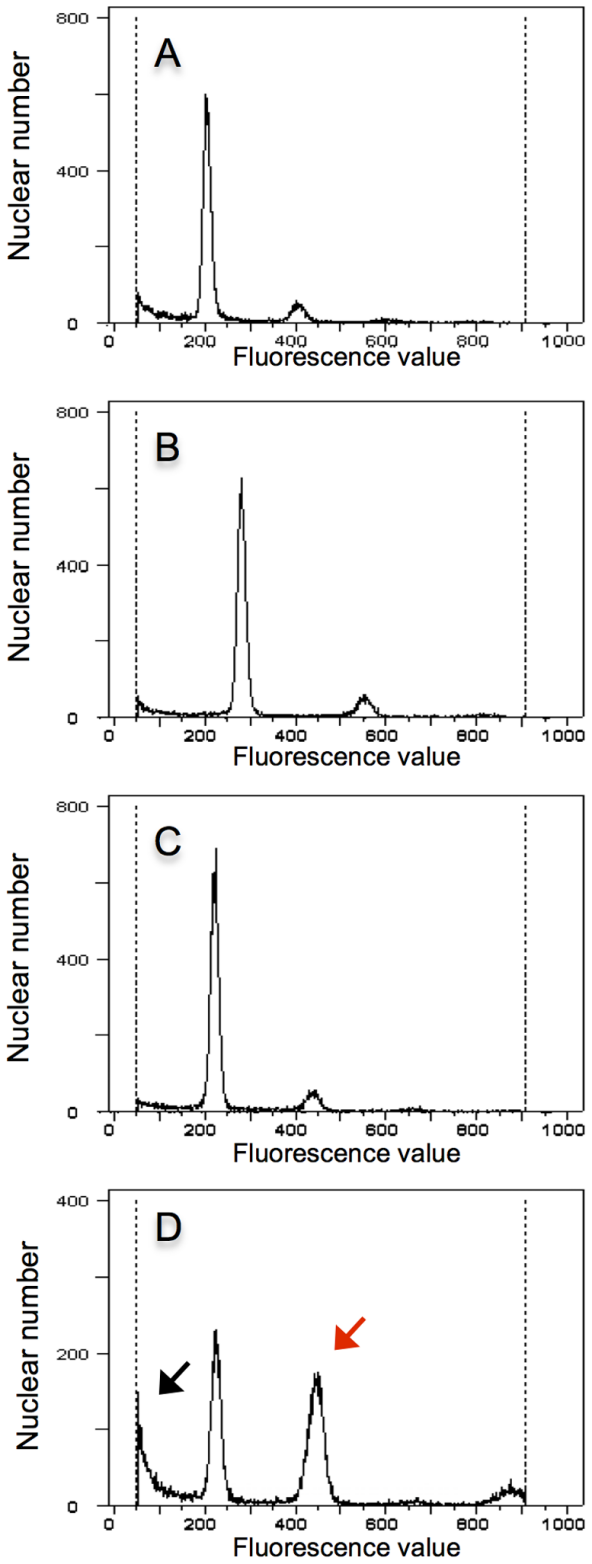
Histograms indicating extent of hybridity for hybrid seedlings of *N. sylvestris*×*N. trigonophylla*. The DNA content of 16,000–22,000 nuclei was determined by flow cytometry. (A) A sample of leaf cells from *N. trigonophylla* as the internal standard. The two peaks are characteristic of G1 nuclei and G2/M nuclei. (B) The DAPI fluorescence values of the G1 peak for *N. sylvestris* were different from that of *N. trigonophylla*. (C) The G1 peak of a viable hybrid plant was intermediate between the two parents. (D) The G1 peak of a vitrified hybrid seedling was intermediate between parents. SubG1 peaks (black arrows) indicating nuclear fragmentation, with lower fluorescence values than that of the G1 peak appeared and the number of nuclei included in the G2/M peak of vitrified hybrids (red arrow) was higher than that of viable hybrid plants. In addition, another G2/M peak was apparent at a fluorescence value of 870.

We used flow cytometry to clarify the extent of hybridity of the vitrified seedlings. The G1 peak of a viable seedling was intermediate between that of its parents, with a fluorescence value of 220 (Figure 6A-C), and the G1 peak of a vitrified seedling appeared at the same fluorescence values as that of a viable seedling (Figure 6C and D). Based on these results, we proved that the vitrified seedlings were true hybrids. However, the number of nuclei included in the G2/M peak of vitrified hybrids was greater than that of viable hybrids (Figure 6D, red arrow). In addition, another G2/M peak was expressed at a fluorescence value of 870 (Figure 6D). These results signify that in the G2/M peak of the vitrified hybrid plants (Figure 6D, red arrow), a G1 peak corresponding to an allotetraploid was included.

The subG1 peak (corresponding to fragmented nuclei) with lower fluorescence values than that of the G1 peaks also appeared for the cross *N. trigonophylla*×*N. tabacum* (Figure 6D, black arrow). This characteristic suggests that nuclear fragmentation progressed with vitrification.

### Viable Hybrid Seedlings of *N. trigonophylla*×*N. tomentosiformis*

**Figure 7:**
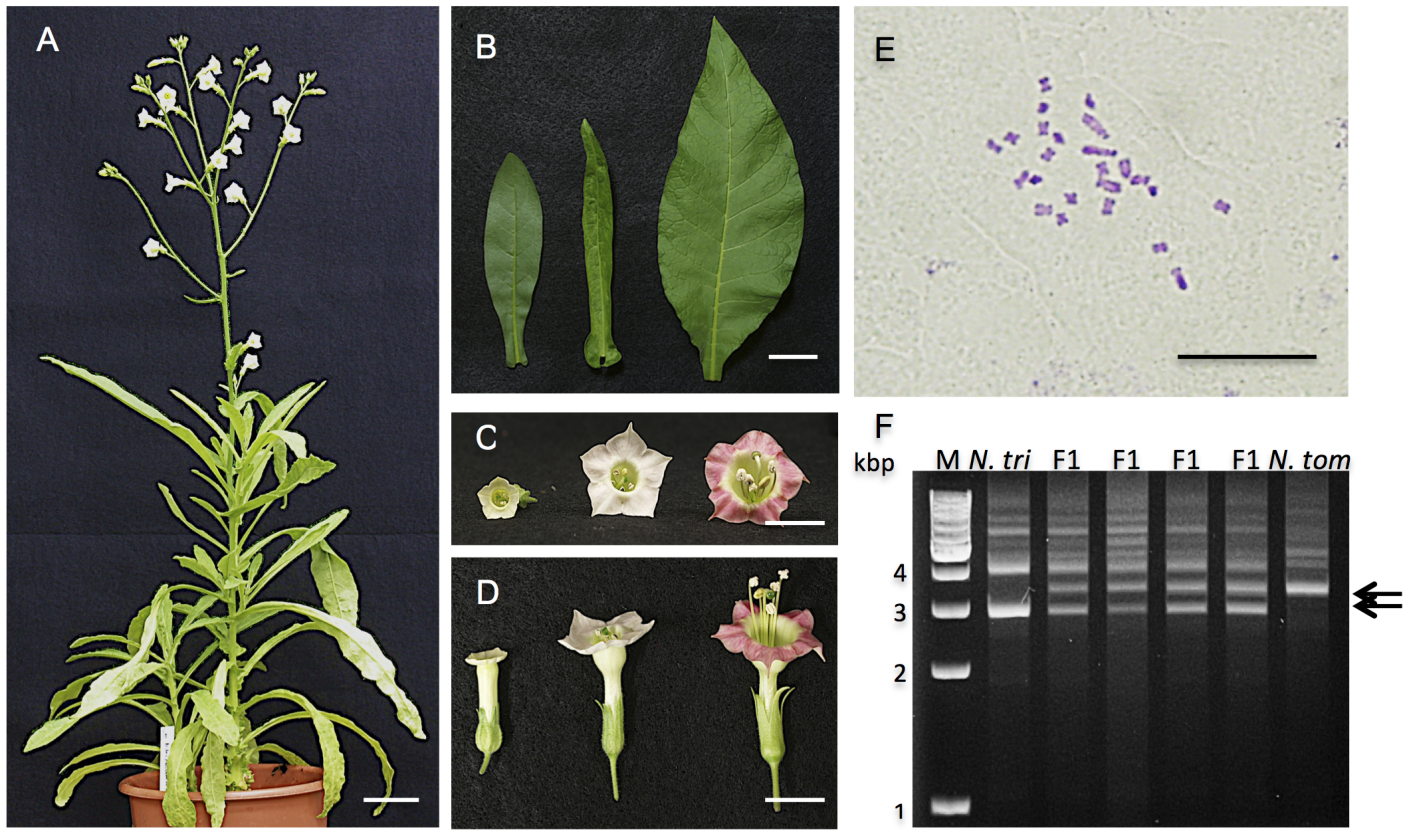
A hybrid of *N. trigonophylla*×*N. tomentosiformis*. (A) Shape of a hybrid plant that grew to maturity and flowered. (B) Leaves of *N. trigonophylla*, a hybrid and *N. tomentosiformis* (left to right). (C and D) Flowers of *N. trigonophylla*, a hybrid and *N. tomentosiformis* (left to right). (E) Image of root tip cell of a hybrid plant, showing the number of chromosomes. (F) Confirmation of hybrid formation by RAPD analysis using primer OPE-14. The bands characteristic of both parents are indicated by arrows. M, size marker OneSTEP Ladder 1kb (1–10 kbp). Scale bars are 10 cm (A), 3 cm (B), 1 cm (C and D) and 100 µm (E).

Results of conventional cross-pollination between *N. trigonophylla* and *N. tomentosiformis* are shown in Figure 7. Hybrid seedlings were obtained approximately 3 weeks after pollination. Hybrid seeds from *N. trigonophylla*×*N. tomentosiformis* crosses were sown in 1/2x MS medium and germinated at 28°C. The hybrid seedlings germinated well, and all of the hybrid seedlings were viable at 20 DAG. These seedlings grew to maturity and flowered (Figure 7A). The morphological characteristics of hybrid plants were uniform and intermediate in appearance between those of the parents, including the leaf shape and flower shape (Figure 7B-D). The chromosome number of the hybrid plants was 24, the sum of the number of haploid chromosomes of the parents (Figure 7E). RAPD patterns for hybrid plants showed several clear bands characteristic of the parents using several primers, indicating that they are true hybrids (data not shown). RAPD patterns obtained with the primer OPE-14 are shown in Figure 7F.

## Discussion

**Figure 8:**
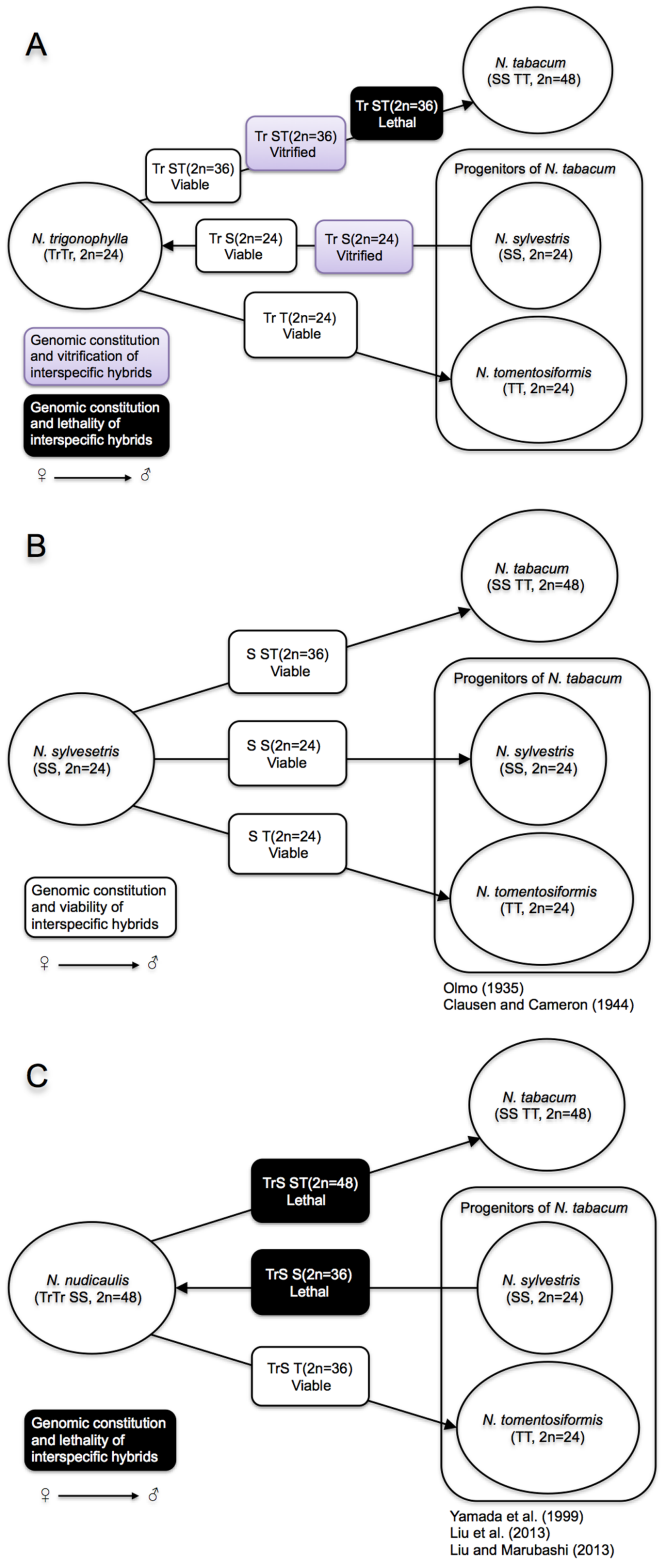
Scheme showing genomic factors responsible for hybrid lethality or vitrification in interspecific crosses involving *N. nudicaulis* and its progenitors.

Vitrification is a morphological and physiological disorder affecting in vitro regenerated plants, and is promoted by several environmental factors including low gelling agent concentrations, liquid media, high relative humidity, high water potential in the medium, excess auxin or cytokinin, low agar concentration, high ammonium levels and high airtightness of the vessel [51]–[56]. However, vitrification occurring in interspecific crosses does not appear to have been reported previously. Though the focus of the paper was on hybrid lethality, a connection to vitrification was one of our findings. We observed vitrified hybrid seedlings from *N. trigonophylla*×*N. tabacum* when they were cultured in MS medium (in the absence of added plant growth regulators), and the number of vitrified hybrids was approximately 50% of the total (Table 1). In *Nicotiana*, hybrid lethality is classified into five types based on the external phenotype: Type I, browning of shoot apex and root tips; Type II, browning of hypocotyls and roots; Type III, yellowing of true leaves; Type IV, formation of multiple shoots; and Type V, fading of shoot color [7], [8]. To our knowledge, of 26 species in section *Suaveolentes*, 22 have been crossed with *N. tabacum* and viability of hybrid seedlings has been reported. Among these crosses, hybrid lethality (Type II or V) is observed in the hybrid seedlings of 20 wild species and *N. tabacum*. In two other crosses, hybrid seedlings are viable [2]. However, we found that the morphology of hybrid plants of *N. trigonophylla*×*N. tabacum* is nonuniform (Figure 8A). In several previous reports, it was reported that the phenotype of hybrid seedlings in reciprocal crosses was the same [8]. Since in crosses between section *Suaveolentes* species and *N. tabacum*, all hybrid seedlings either express hybrid lethality or do not, we speculate that *N. trigonophylla* has causative genomic factors affecting the phenotype of hybrid seedlings.

Similar observations of vitrification occurred in a cross between *N. trigonophylla* and *N. sylvestris*, but not in a cross of *N. trigonophylla*×*N. tomentosiformis*. These results indicate that the vitrification in the interspecific cross *N. trigonophylla*×*N. tabacum* is genetically controlled, and that the S subgenome of *N. tabacum* controls it (Figure 8A).

Section *Repandae* formed from a single hybridization event between relatives of extant *N. sylvestris* and *N. trigonophylla*, the respective maternal and paternal progenitors, producing four allopolyploids: *N. nudicaulis*, *N. repanda*, *N. stocktonii* and *N. nesophila* [17], [32], [33]. When *N. sylvestris* was crossed with *N. tabacum*, viable hybrids were obtained (Figure 8B). On the other hand, in the cross *N. nudicaulis*×*N. tabacum*, hybrid lethality was expressed and controlled by the S subgenome of *N. tabacum* (Figure 8C); the genomic factor related to vitrification is also in the S subgenome (Figure 8A). Based on these results, we inferred that the genomic factor in *N. nudicaulis* controlling hybrid lethality in the cross *N. nudicaulis*×*N. tabacum* is not related to the maternal progenitor, *N. sylvestris*, but is inherited from the paternal progenitor, *N. trigonophylla*. Although in the cross *N. trigonophylla*×*N. tabacum*, approximately 50% of hybrid seedlings showed vitrification, and hybrid lethality is expressed in the cross *N. nudicaulis*×*N. tabacum*, both *N. nudicaulis* and *N. trigonophylla* interact with the same subgenome of *N. tabacum* (the S subgenome) to control hybrid lethality or vitrification. Moreover, when we confirmed the hybridity of the vitrified seedlings of both *N. trigonophylla*×*N. tabacum* and *N. sylvestris*×*N. trigonophylla* using flow cytometry, we detected characteristic patterns of subG1 peaks with lower fluorescence values than observed for G1 peaks, indicating nuclear fragmentation (Figures 3C-E and 6C, black arrows); the fluorescence of these subG1 peaks increased in the seedlings of *N. nudicaulis*×*N. tabacum* as they progressed through the stages of apoptotic cell death [38]. Thus, these results indicate that vitrification and hybrid lethality share the same hallmark of apoptosis, namely nuclear fragmentation. Furthermore, based on these results, we infer that vitrification is related to hybrid lethality.

Recent advances have highlighted the ubiquity of whole-genome duplication (polyploidy) in angiosperms; although global analyses of genome size reveal a trend towards DNA loss subsequent to polyploidy (genome downsizing), increases in genome size also occur [34], [57]. In section *Repandae*, there is considerable departure from additivity in seven transposable element families, typically through deletion, especially for transposable elements derived from the *N. obtusifolia* parent [35]. Moreover, Renny-Byfield et al. [58] presented evidence that loss of high-copy-number repeats in the genome of *N. nudicaulis*, some of which are Ty3/Gypsy retroelements, contributed to a reduction of approximately 14% in genome size, similar to the 19% estimated using Markov chain Monte Carlo approaches. From these reports, we speculate that when *N. nudicaulis* was formed from the hybridization of *N. sylvestris* and *N. trigonophylla* approximately 5 million years ago, several genomic factors determining phenotypes of hybrid seedlings were inherited from *N. trigonophylla*. Subsequently, genome downsizing and various recombination-based processes took place. Some of the causative genomic factors were lost and some of them became genomic factor(s) controlling hybrid lethality in extant *N. nudicaulis*.

Currently, we are attempting to perform crosses of monosomic lines of *N. tabacum* and *N. trigonophylla* or *N. nudicaulis* to determine the chromosome(s) of *N. tabacum* responsible for vitrification of *N. trigonophylla*×*N. tabacum* and hybrid lethality of *N. nudicaulis*×*N. tabacum*. In the present study, we found that fragmentation of nuclei was accompanied by vitrification during progression of lethal symptoms in hybrids; therefore, we plan to use additional cytobiological approaches to determine whether apoptotic cell death accompanies vitrification.

Such an approach may provide important information revealing the relationship between vitrification and hybrid lethality. Furthermore, we expect these planned experiments will contribute to clarifying the origins of genomic factor(s) in *N. nudicaulis* controlling hybrid lethality in interspecific hybrids between *N. nudicaulis* and *N. tabacum*. This knowledge will likely provide a better understanding of hybrid lethality as a reproductive barrier, which would have broad applications in agriculture.
